# Sequencing through hyperexpanded Friedreich’s ataxia-GAA repeats by nanopore technology: implications in genotype–phenotype correlation

**DOI:** 10.1093/braincomms/fcad020

**Published:** 2023-03-29

**Authors:** Bharathram Uppili, Pooja Sharma, Istaq Ahmad, Shweta Sahni, Vivekanand Asokachandran, Anil B Nagaraja, Achal K Srivastava, Mohammed Faruq

**Affiliations:** Genomics and Molecular Medicine, CSIR-Institute of Genomics and Integrative Biology (CSIR-IGIB), Delhi 110007, India; Academy for Scientific and Innovative Research, Ghaziabad 201002, India; Genomics and Molecular Medicine, CSIR-Institute of Genomics and Integrative Biology (CSIR-IGIB), Delhi 110007, India; Academy for Scientific and Innovative Research, Ghaziabad 201002, India; Neurology Department, Neurosciences Centre, All India Institute of Medical Sciences, New Delhi 110029, India; Neurology Department, Neurosciences Centre, All India Institute of Medical Sciences, New Delhi 110029, India; Genomics and Molecular Medicine, CSIR-Institute of Genomics and Integrative Biology (CSIR-IGIB), Delhi 110007, India; Academy for Scientific and Innovative Research, Ghaziabad 201002, India; All India Institute of Medical Sciences, Jodhpur, Rajasthan 342001, India; Neurology Department, Neurosciences Centre, All India Institute of Medical Sciences, New Delhi 110029, India; Genomics and Molecular Medicine, CSIR-Institute of Genomics and Integrative Biology (CSIR-IGIB), Delhi 110007, India; Academy for Scientific and Innovative Research, Ghaziabad 201002, India

**Keywords:** FRDA, long-read sequencing, trinucleotide repeats, GAA repeats

## Abstract

Friedreich’s ataxia, an autosomal recessive disorder, is caused by tandem GAA nucleotide repeat expansions in intron 1 of the frataxin gene. The GAA repeats over 66 in number are considered as pathogenic, and commonly occurring pathogenic repeats are within a range of 600–1200. Clinically, the spectrum of features is confined mainly to neurological tissues; however, cardiomyopathy and diabetes mellitus have been reported in 60 and 30% of the subjects, respectively. The accurate detection of GAA repeat count is of utmost importance for clinical genetic correlation, and no study so far has attempted an approach that is of high-throughput nature and defines the exact sequence of GAA repeats. Largely, the method for detection of GAA repeats so far is either through the conventional polymerase chain reaction-based screening or Southern blot, which remains the gold standard method. We utilized an approach of long-range targeted amplification of *FXN*-GAA repeats using Oxford Nanopore Technologies MinION platform for accurate estimation of repeat length. We were able to achieve successful amplification of GAA repeats ranging from ∼120 to 1100 at ∼2600× mean coverage. The total throughput achievable through our protocol can allow for screening of up to 96 samples per flow cell in less than 24 h. The proposed method is clinically scalable and deployable for day-to-day diagnostics. In this paper, we demonstrate to resolve the genotype–phenotype correlation of Friedreich’s ataxia patients with better accuracy.

## Teaser

Uppili *et al*. described a simple and efficient method to sequence full length hyperexpanded GAA repeats of Friedreich’s ataxia loci (*FXN*). Long-range PCR amplification of *FXN*-GAA repeats followed by sequencing on nanopore (Oxford Nanopore Technologies) provided an accurate GAA length estimation and a fair estimate about the repetitive sequence content of GAA repeats.

## Graphical Abstract

**Graphical Abstract:**
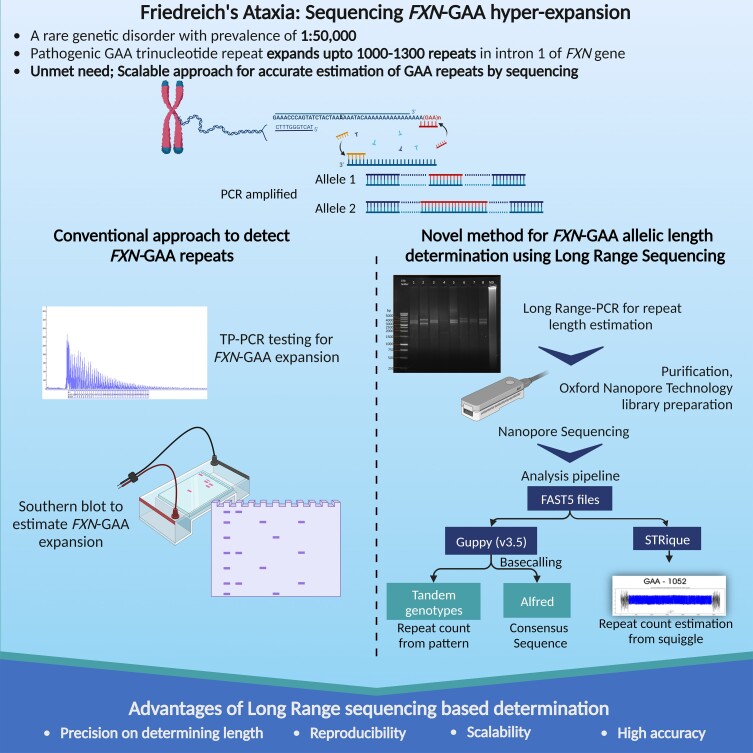

## Introduction

Friedreich’s ataxia (FRDA) is an autosomal recessive disorder that affects neurological (spinal cord, sensory nerves and cerebellum) and extra-neuronal systems (heart and pancreas). Disease onset usually occurs before 25 years of age. The causal mutation is a hyperexpansion of GAA repeats within intron 1 of the frataxin gene (*FXN*) gene on chr9q21.11. The GAA repeat length varies in healthy subjects from 5 to 33. Alleles with GAA repeats greater in number than 66, up to 1700 repeats, represent full length pathogenic alleles.^1,2^ The most common GAA repeat length observed in patients is between ∼600 and 1200. The cardinal clinical symptoms and signs of FRDA include gait ataxia, dysarthria, peripheral neuropathy, kyphoscoliosis and pes cavus, while ∼60% of the patients develop hypertrophic cardiomyopathy and nearly one-third of the patient manifest diabetes mellitus. Nearly 25% of all FRDA cases do not subscribe to the essential diagnostic criterion for age of onset before 25 years as defined by Harding^3^ and are referred to as the late-onset FRDA.^4^ Atypical features in late-onset FRDA are defined by clinically milder disease phenotypes with a much slower disease progression attributed to the smaller size of the expanded repeat on GAA1.^5,6^

The method of detection of *FXN*-GAA repeats is through conventional polymerase chain reaction (PCR), coupled with triplet repeat-primed PCR. Triplet repeat-primed PCR is helpful in screening; however, Southern blot is the gold standard method to detect the GAA length. Precise determination of accurate GAA length holds considerable importance because the observed clinical variability in FRDA remains largely unexplained. Some of the pertinent clinical and technical issues that warrant alternate and advanced tools to detect not only the GAA repeat length but also the occurrence of repeat interruption are as follows: (i) the determination of sequence configuration of GAA track would allow its correct interpretation if its purity can be ascertained vis-a-vis the interrupting sequences; (ii) the GAA sequence determination would allow further understanding of the genotype–phenotype correlation; (iii) it would enable elucidation of the relation between late-onset FRDA and interrupted GAA sequences and (iv) Southern blot is a low-throughput and time-intensive technique for determination of GAA length. Sequence variation within the GAA repeat region has been reported to affect the phenotypic variability in FRDA, especially in cases with late onset of disease.^5,6^ The aforementioned issues underscore the need for development and deployment of a novel approach to accurately determine the GAA length via next-generation sequencing.

This study reports a comprehensive and simple protocol used for accurate determination of GAA repeats using Oxford Nanopore Technologies (ONT). We were successful in diagnosing a suspected case of late-onset spinocerebellar ataxia (SCA) phenotype as FRDA and able to correctly estimate the length of *FXN*-GAA repeats using long-read sequencing (LRseq). We were successful in estimating the length of GAA repeat regions within the range of 120–1100 repeats with precise accuracy. Furthermore, sequencing-based repeat length estimation for FRDA subjects, with varying repeat numbers and age at onset, allowed better assessment of genotype–phenotype correlation.

## Methods

### Case selection and sample screening

A multigenerational family with two of its members clinically diagnosed for late-onset SCA phenotype was recruited for genetic investigations (2 affected and 10 unaffected, at-risk individuals). Genomic DNA was isolated from blood specimens using the standard protocol. DNA concentration and purity were checked through the NanoDrop spectrophotometer. Genomic DNA of suspected cases from these kindred were subjected to common SCA mutation screening for SCA1, SCA2, SCA3, SCA6, SCA7, SCA12 and SCA17 using fluorescently labelled primers, all of which were negative. FRDA-GAA expansion screening was done through triplet repeat-primed PCR,^7^ which showed presence of *FXN*-GAA expansions in both affected and unaffected individuals. Long-range PCR (LR-PCR) was then performed on samples positive for *FXN*-GAA expansion to confirm the allelic status for length and homozygosity. The details of primers and reaction conditions are provided in Supplementary material. The results were confirmed by observation of DNA amplicon bands via agarose gel electrophoresis (Supplementary Fig. 2).

### ONT library preparation and sequencing

Accurate estimation of *FXN*-GAA repeat length for FRDA patients was performed using LRseq of the *FXN*-GAA locus using ONT. Additional FRDA patient samples (*n* = 09) with varying repeat lengths as confirmed by LR-PCR and healthy control (*n* = 1), from our in-house DNA repository, were also included in the study. Initial amplification of the target *FXN*-GAA locus was performed using LR-PCR method as described above. PCR amplicons were checked on 1% agarose gel with 1 Kb DNA ladder (Promega, Catalog no.: G5711). The amplified PCR products were ranged in size from 1.4 to 4.5 kbp depending on the repeat length. Amplicons were purified using AMPure Beads (Beckman Coulter, Catalog no: A63881) in 1.6× ratio by volume, for further use in library preparation and sequencing.

Around 400 ng of purified PCR amplicons were used as input for library preparation. The library was prepared following the ONT native barcoding protocol using EXP-NBD196 and SQK-LSK109 kits, in accordance with the manufacturer’s instructions. The DNA amplicons were end repaired and dA-tailed using NEBNext FFPE DNA Repair Mix [New England Biolabs (NEB), Ipswich, USA] and NEBNext End repair/dA-tailing (NEB) (catalog no. E7546). Native barcodes were ligated to the end repaired DNA using NEB Blunt/TA Ligase Master Mix (SQK-LSK109). Adaptor ligation to the pooled barcoded library was performed using NEBNext^®^ quick ligation module (NEB E6057). The final library was purified with AMPure Beads in 0.4× ratio of the reaction volume and ONT short fragment buffer (SFB-SQK-LSK109). Around 70 ng of the purified final libraries were loaded onto an ONT-MinION flow cell (R9.4.1) and sequenced on ONT-MinION Mk1C device for a duration of around 16 h.

### Sequence analysis and repeat genotyping

Guppy basecaller (version 3.5.2) was used to demultiplex the raw fast5 files into FASTQ files.^8^ Alignment to the reference human *FXN* gene sequence (GRCh37—chr9:71650479–71693993) was performed with Minimap2 (v 2.1).^9^ The resulting bam file was sorted and indexed using SAMtools 2 (v 1.9).^10^ STRique was then used for analysis of *FXN*-GAA repeat lengths.^11^ After indexing the fast5 files, the reference sequence having the repeat region was used to identify the repetitive squiggle pattern. Repeat count per each read, for all the samples, was calculated using the HMM model of the STRique (r9_4_450bps.model). For each sample, the repeat length value with the highest number of supporting reads (mode) for each allele was considered as the STRique reported *FXN*-GAA repeat length. Alfred (v 0.2.1) was used to generate the consensus sequence for determination of the repeat composition and to check for the presence of interruptions.^12^ Reads having ±20 repeats from the estimated STRique count were used for creating the consensus sequence. To cross-validate the presence of interruptions within the repeat sequence, consensus sequence was created using reads from both positive and negative strands. Presence of small insertions was evaluated using tandem repeat finder (v 4.09) and manually checked and compiled.^13^ Data plotting and visualization were performed using the ggplot2 and dplyr packages from R.

To benchmark results from STRique, we used Tandem-genotypes^14^ to re-estimate the repeat length from nanopore raw FASTQ. LAST tool^15^ was used for preparing reference without repeat masking (lastdb). Substitution and gap rates between reads and genome were determined using LAST-train, later, aligned the raw reads with the reference sequence using lastal. The resulting .maf file and a bed file with the expected repeat region were used as an input for Tandem-genotypes tool to quantify repeat expansions with predicted copy number changes per repeat for each allele (option -o2).

### Statistical analysis

The correlation between different methods used to determine repeat length was done using the Pearson correlation method. A linear regression analysis was carried out for allele length and age at onset from STRique and LR-PCR data. Correlation matrix and scatter plots were made using R 4.0 (ggplot2 v3.3.6) and Python 3.9.7 (seaborn v0.11.2).

## Results

### Clinical details of the subjects

#### Case report of the kindred with late-onset cerebellar ataxia

A 69-year-old male [Pedigree chart, Supplementary Fig. 1, IV:7; (GOS10454)], born to third degree consanguineous parents, who was apparently healthy till 35 years of age, developed frequent falls while walking that progressed to walking with support by 45 years. At the time of writing this report, the proband was wheelchair bound. On general examination, tachycardia and stage-I hypertension were observed while the subject was on medications. Scoliosis was noted. Neurological examination revealed presence of cerebellar gait ataxia, intention tremors, dysdiadochokinesia and brisk reflexes. No nystagmus was observed. The subject did not have any other relevant medical or surgical history of importance relevant to the diagnosis. Blood biochemical profile reported normal indices for liver function and renal function tests and serum Vitamin E and Vitamin B12 levels within normal range. SCA1, SCA2, SCA3, SCA6, SCA7, SCA12 and SCA17 CAG repeat testing was negative. Next-generation sequencing for OMIM reported via ∼8000 gene panel test was inconclusive for any pathogenic mutations (done commercially). The family reported a second affected individual IV: 3, a cousin sister of the proband [Supplementary Fig. 1, IV:3; (GOS10455)] of age 55 years, a female, sixth born to third degree consanguineous parents. She developed frequent falls from the age of 30 years and difficulty in balancing while standing and walking. Neurological examination revealed features suggestive of cerebellar ataxia, and no other system was found to be affected with abnormalities. A third affected male member of the same family [Supplementary Fig. 1, V:2; (GOS10456)], a relative of the proband, 48 years, reported symptoms of progressive diminution of vision from the age of 30 years. He was clinically diagnosed with retinitis pigmentosa and had no other reported neurological abnormalities including cerebellar ataxias.

After multiple unsuccessful attempts for clinical diagnosis at multiple clinical centres and neurology clinics, the above patients were referred for genetic testing of SCAs. The three affected cases and other family members were enrolled in the study for further genetic evaluation. Dominant SCAs were ruled out, and NGS-based tests were inconclusive. Following a heuristic approach, *FXN*-GAA repeat expansion screening was performed primarily to rule out common mutations before dwelling into complicated genetic investigations such as whole genome sequencing. Triplet repeat-primed PCR-mediated *FXN*-GAA screening showed positive results for the two affected individuals and other family members. To rule out the possibility of *FXN*-GAA expansion in a carrier state, LR-PCR was performed to detect both the alleles. Results of LR-PCR confirmed the presence of biallelic expansion for *FXN*-GAA repeats in the two affected subjects (proband-IV:7 and his relative-IV:3) with an estimated repeat number of 270/1004 and 287/1037 (±30) (Supplementary Fig. 2). Remaining at risk family members were carriers of *FXN*-GAA repeat expansion with significant variability in length of both the alleles among the family members.

### Clinical details of FRDA subjects selected for length estimation of GAA repeats by ONT sequencing

Nine other cases of FRDA with varying age at onset (2–44 years) and LR-PCR-based *FXN*-GAA length between 120 and 1200 were also included in the study (Table 1). Among five subjects with approximated GAA repeat configuration (allele1/allele2) of >700/>1100, age at disease onset varied from 2 (AT-3285) to 15 years (AT-1967). The *FXN*-GAA length difference between allele1 and allele2 in these subjects was ∼300–350. Three subjects (including two cases described above) had onset after the age of 30 years and had allele1/allele2 difference of ∼700 GAA repeats. Except for one patient (AT-866), seven subjects with age at onset ≤16 years had abnormal echocardiography findings with left ventricular hypertrophy and five of the patients had abnormal nerve conduction findings (Table 1).

**Table 1:** Demographic details of FRDA patients describing clinical and genetic features for GAA length in *FXN*

Parameters	AT-3285	AT-866	GOS-3932	AT-2001	AT-1967	GOS-142	GOS-322	GOS-9443	GOS-10455	GOS-10454	GOS-4200
Age/gender	7/F	16/M	15/M	16/M	18/F	19/M	19/M	20/M	55/F	69/M	52/F
Age at onset in years	2	10	12	13	15	16	16	18	30	35	44
STRique-GAA	872/1056	832/1106	708/900	759/915	728/946	652/849	608/728	791/824	282/1062	257/1055	125/876
RP-PCR	867/1200	866/1183	870/1204	704/1037	833/1200	654/904	620/1054	870/870	287/1037	270/1004	120/870
First symptom	GA	GA	GA	GA	GA	GA	GA	GA	GA	GA	GA
Cardiac symptoms	+	−	+	+	+	+	−	−	−	Tachycardia	−
Dysarthria		+ (AO:11yrs)	+ (AO:12yrs)	+ (AO:15yrs)	−	+	+	+ (AO:20yrs)	−	NR	+ (AO:47yrs)
Nystagmus		−	+	−	+	−	+	−	−	−	+
DTR (areflexia)	NR	+	+	+	+	+	+	+	−	Brisk reflexes	NR
Plantar	NR	Extensor	Extensor	Extensor	Flexor	Extensor	Extensor	Extensor	Flexor	Unequivocal	Flexor
Pes cavus	+	NR	+	+	NR	NR	NR	−	−	−	−
Scoliosis	−	NR	+	+	+	NR	+	+	−	+	NR
Extrapyramidal signs	NR	NR	−	NR	−	Bradykinesia	Bradykinesia and facial kinesis	Bradykinesia	−	−	Bradykinesia
ECHO	Non-obstructive HCM	Normal	LVH	Mild -LVH	HCM	Mild conc. LVH	LV-enlarged	Normal	NR	Normal	Normal
EPS	SMN axonal	Normal	SN	SN	SN	NR	Abnormal SSR	NR	NR	Normal	NR
CT/MRI brain	NR	MRI- cerebellar atrophy	MRI-brain normal	CT- normal	Normal study	NR	MRI-normal study	CT- normal, MRI- normal	NR	MRI-diffuse cerebellar atrophy.	CT-cerebral atrophy, MRI-b/l frontal parietal deep white matter changes
Spinal cord Atrophy		−	−	−	+	NR	−	NR	NR	−	−

### Targeted FXN-GAA flanking region sequencing on MinION flow cell

The long-range amplification using flanking primer sequences amplified sample specific PCR products of varying length ranging from 1500 to 4500 base pairs (Supplementary Fig. 2). The PCR products of length ∼1.5 kb carry small normal GAA repeats^7–20^ while fragments of 2.2 kb carry ∼200 GAA, likewise, fragments of size ∼4 kb correspond to ∼1100 GAA repeats (Supplementary Table 1).

Following LR-PCR-based amplification, amplicons corresponding to *FXN*-GAA repeat flanking region for 11 FRDA patients and 1 healthy control were sequenced by LRseq. Amplicons from 11 FRDA subjects corresponded to GAA (allele1/allele2) repeat numbers in the range of 120–1200 while *FXN*-GAA amplicons from healthy control had 8/8 repeat configuration. We obtained an average of 5 k reads per sample that mapped to the target region, with a mean coverage of ∼2600× (607–5544×). The data QC showed the maxima of sequenced read length corresponding to 4 kb length with some non-specific longer products. The percent GC content of the sequenced reads ranged from 42 to 45% and read mapping percentage per sample varied from 12 to 47% (Supplementary Table 2).

### GAA repeat length and repeat configuration assessment and clinical implications

**Figure 1:**
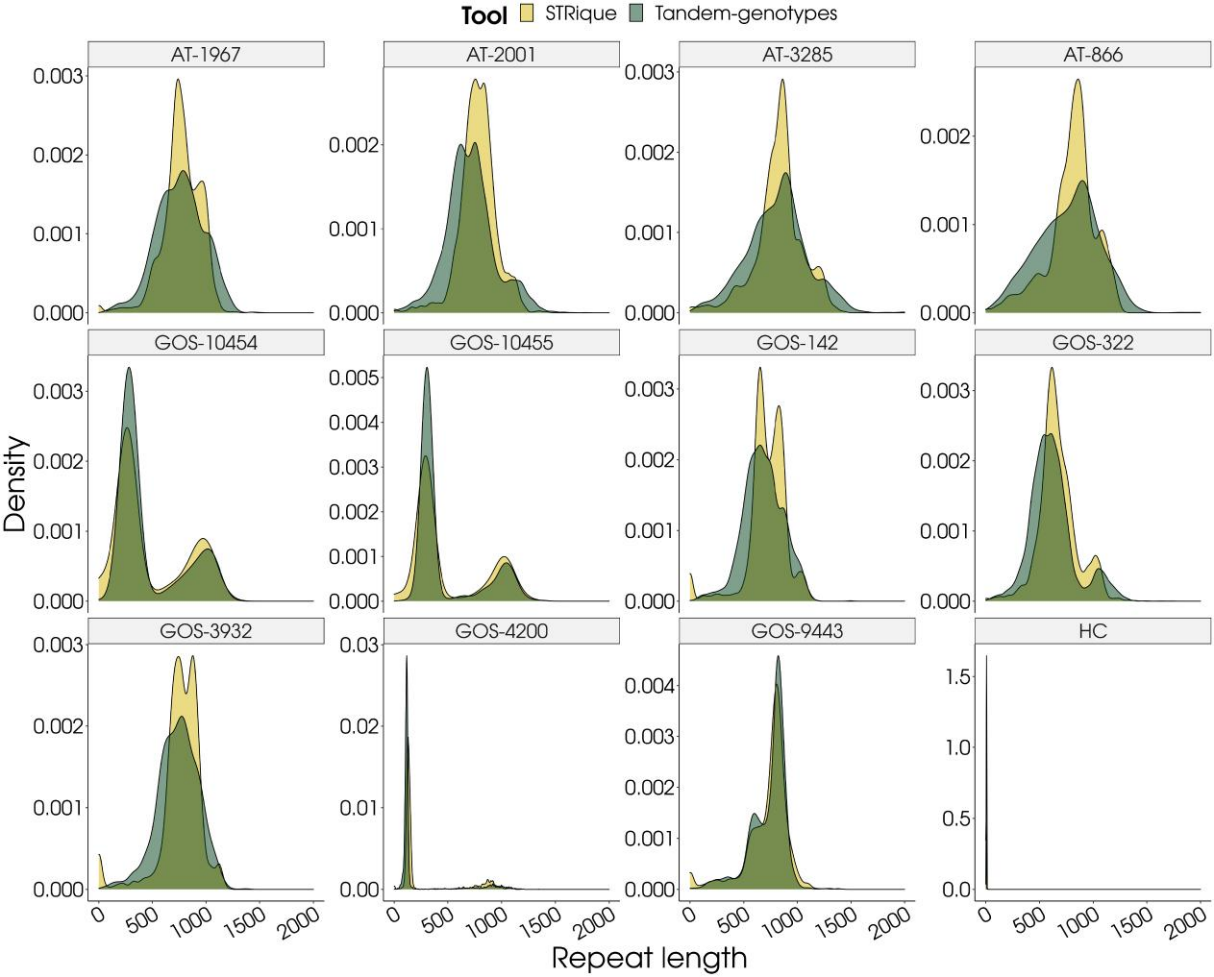
**GAA repeat length assessment by tandem genotype and STRique.** Histogram showing distribution of various GAA repeat reads (length) and their abundance as shown for tandem genotyper and STRique tools. Within sample variability in GAA length is indicative of somatic mosaicism.

**Figure 2:**
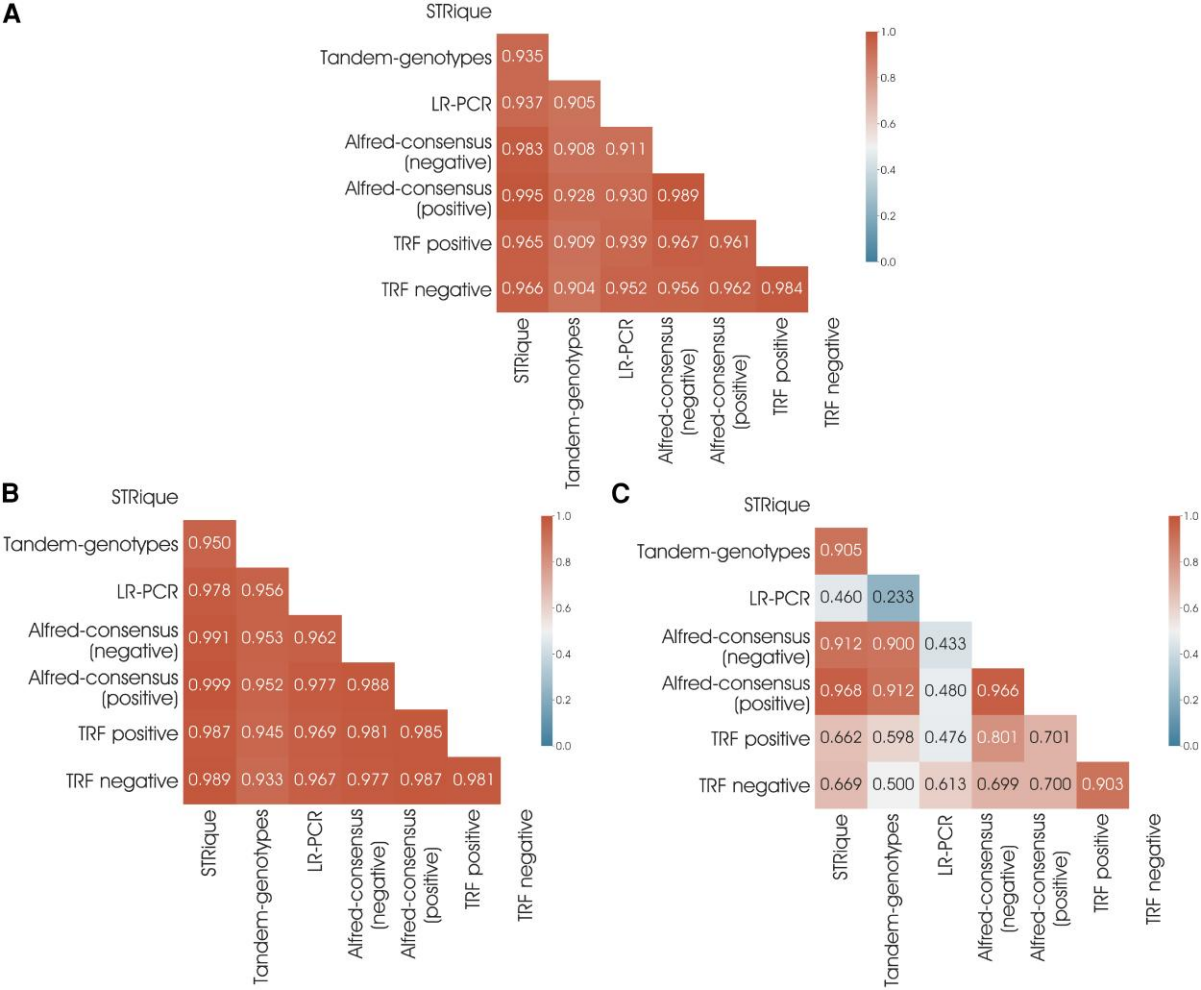
**Between tool correlation matrices of the obtained GAA repeat length.** Pearson correlation coefficient *r* values (matrix) for (**A**) combined GAA length (allele1 and allele2) estimation between various methods, (**B**) for GAA allele 1 and (**C**) for GAA-allele 2. Allele 1 had a more deterministic influence on overall correlation between methods than allele 2, even though STRique and LR-PCR had a *r* value of 0.94 overall correlation (Fig. 2A), while allele 2 had a lesser correlation with *r* value of 0.4 between the two techniques (Fig. 2B and C).

The length estimation of GAA repeats was done using STRique and tandem genotype, and sequence configuration assessment was done via consensus sequence calling. For each sample, STRique analysis output provided individual reads with GAA repeats count along with the strand information. STRique utilized repeat count estimation from squiggles of nanopore reads (Supplementary Fig. 3). STRique output reads with the maximum mode were considered to call for biallelic definition to obtain the GAA repeats for each sample (Fig. 1). *FXN*-GAA repeat length calculation as estimated by STRique for all samples was in the range of 125–1106 (Table 2). The repeat length estimation for all samples as done by the tandem genotype was in the range of 115–950. The average difference between allele sizes from both the tools was +88 repeats, where tandem genotype estimated slightly lesser length than expected in three of the samples; the differences were around 300 repeats. Though the overall correlation between STRique and LR-PCR was 0.94 (r) (Fig. 2A), the effect of allele 1 was deterministic of overall correlation than allele 2 while allele 2 showed a weaker correlation of 0.4 (r) between the two methods (Fig. 2B and C). We also computed the GAA length by manually inspecting GAA repeats in Alfred-generated consensus sequences separately for positive (GAA) and negative (CTT) strand. Manual method-based GAA length almost correlated completely (*r* = 1) with STRique than tandem repeat finder (*r* = 0.97). Thus, the length estimates are accurate with STRique for GAA repeat length estimation and hence further used for the genotype–phenotype correlations.

**Table 2:** GAA length estimation by targeted LRseq on nanopore using STRique and tandem genotype

		(+) Strand					(−) Strand					Computational tool analysis	
				Tandem repeat finder					Tandem repeat finder				
Sample-ID	allele	Alfred-GAA	Read counts	Copy number	Percent matches	Percent indels	Alfred-GAA	Read counts	Copy number	Percent matches	Percent indels	STRique-GAA	Tandem genotypes-GAA
AT-3285	A1	823	27	824.7	94	5	918	222	920.7	96	3	872	609
	A2	1007	12	975.7	91	8	1127	32	1121.7	97	2	1056	950
AT-866	A1	807	12	772.7	93	6	864	70	861.7	98	1	832	524
	A2	1067	2	994.7	86	12	1158	24	1155.7	99	0	1106	931
GOS-3932	A1	707	12	689.7	94	5	731	147	733.7	97	2	708	613
	A2	845	25	827.7	92	6	934	95	920.7	98	1	900	862
AT-2001	A1	751	50	721.7	93	6	779	101	803.7	95	3	759	572
	A2	847	70	807.7	92	7	847	50	921.7	97	1	915	829
AT-1967	A1	733	14	698.7	91	8	769	227	793.7	93	5	728	602
	A2	921	8	901.7	89	10	1006	77	1037.7	96	2	946	901
GOS-142	A1	652	14	642.7	95	4	690	227	690.7	97	2	652	573
	A2	788	19	770.7	91	8	880	134	880.7	98	1	849	822
GOS-322	A1	603	153	599.7	89	9	654	586	655.7	91	8	608	490
	A2	725	184	922.7	88	11	744	74	1066.7	97	2	728	709
GOS-9443	A1	803	36	803	99	0	834	316	832.7	97	2	791	583
	A2	842	36	842	99	0	850	280	851.7	99	0	824	822
GOS-10455	A1	318	151	318	90	2	305	204	151.3	98	1	282	304
	A2	1071	4	1067	98	1	1110	54	1106.7	99	0	1062	1020
GOS-10454	A1	271	13	136	98	0	293	118	146.8	98	0	257	286
	A2	na	na	na	na	na	1079	14	1075.7	100	0	1055	976
GOS-4200	A1	123	104	122.7	87	9	131	1216	132.7	89	9	125	115
	A2	855	4	815.7	89	10	918	104	916.7	98	1	876	899
HC	A1	8	309	–	–	–	8	567	–	–	–	8	1
	A2	8	309	–	–	–	8	567	–	–	–	8	4

The GAA repeat configuration was checked by manual as well as tandem repeat finder method. In two subjects (GOS10454 and 10455 from the same kindred), the lower allele of size 282 and 257 GAA repeats had interruption of GGAGAA (Supplementary Table 1). Most of the remaining sequences had near similar non-interrupted patterns, and two alleles of IV:7 and IV:3 had purest sequenced GAA/CTT alleles, while other had indels (%) 1–8%. This may represent sequencing artefacts due to the inherent sequencing error probability of the ONT platform^21^.

**Figure 3:**
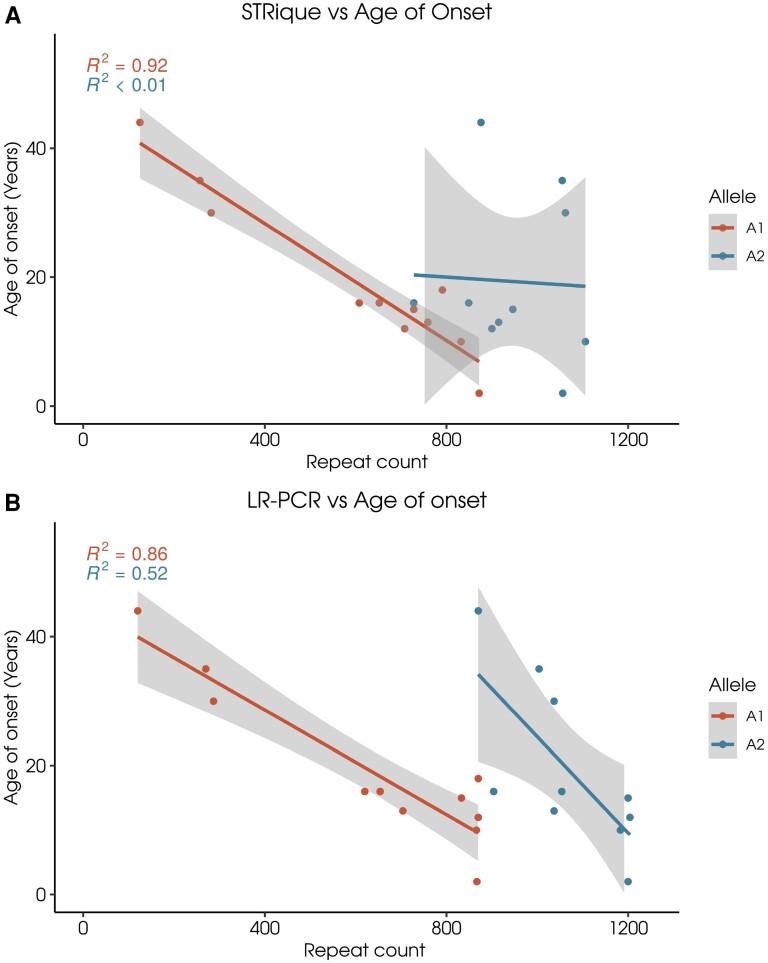
**Correlation of GAA allele 1/allele 2 with age of onset of the FRDA.** Correlation between age of onset and allelic length as estimated by different methods (**A**) STRique and (**B**) RP-PCR.

We observed that *FXN*-GAA length of shorter alleles had the most significant correlation with age at onset (*R*^2^-92%) as measured by STRique versus (*R*^2^-86%) as measured by LR-PCR alone. While the longer GAA allele had shown no correlation with age at onset (*R*^2^< 1% by STRique) versus LR-PCR (*R*^2^-52%) (Fig. 3), this suggests that length accurate estimation of GAA locus improves the genotype–phenotype correlations and would be desirable for further management of clinical phenotype.

## Discussion

Despite their important role in determining the clinical variability in FRDA, the challenging *FXN*-GAA repeat structure investigation has so far precluded large-scale genotype–phenotype correlation studies. As previously studied, the length of GAA repeats in shorter alleles has a significant effect on age at onset in FRDA patients^16–18^ explaining upto 62.9% age at onset variation (in cases of delayed onset FRDA).^18^ Also, the role of interruption in the *FXN*-GAA tract has been intensively studied by various groups and reported to have a significant impact on disease onset or even affect the pathogenesis.^19,20^ LRseq with its promising ability for identification of causative repeat expansions, discernment of repeat configuration including repeat interruptions, repeat length estimation and detection of base modifications, is aptly positioned to overcome these challenges. For the first time, we demonstrate the ability of long-read ONT sequencing in accurately determining the size of an expanded intronic region of up to 1100 *FXN*-GAA repeats in FRDA patients. Using this approach, we solved a case of late-onset cerebellar ataxia in a time- and cost-efficient manner. This was a pilot study with *n* = 12 samples, but this is a scalable approach and may allow for low-cost sequencing of up to 96 samples on a single ONT flow cell. Determination of *FXN*-GAA repeat sequence and configuration, along with precise estimation of repeat length, would allow a proper investigation of difficult to solve late-onset cases of cerebellar ataxias. A similar approach may be utilized for targeted sequencing of other tandem nucleotide repeat (TNR) loci.

While short-read sequencing for detection of repeat-mediated disorders has already progressed much, LRseq as offered by the ONT offers many unprecedented advantages over the former. Besides their rapidly shrinking costs and improved read quality, LRseq platforms provide additional manoeuvring with respect to the bioinformatics analysis pipelines. This is of special significance in diseases attributable to complex expanded alleles with multiple repeat interruptions. LRseq or third-generation sequencing technologies are a promising tool for querying the human genome for the assessment of long tracts of TNR expansion and over the last few years, several novel genes have been identified through LRseq technologies, i.e. single-molecule real-time- and ONT-based platforms. LRSeq has led to the discovery of various new TNR mutations that would otherwise not be detectable through short-read sequencing methods. The discovery of these TNRs has opened new avenues for solving the pathology of challenging neurological disorders, such as benign adult familial myoclonic epilepsy 1 (*SAMD12*)^22^ and neuronal intranuclear inclusion disease (*NOTCH2NLC*).^23^

The shortcomings of the existing approaches along with the unparalleled advantages offered by the ONT-based LRseq make it imperative to deploy this technology. It is very important now to use the same technology for accurate detection of TNR sequences for known disease loci, and efforts made in this direction in the past have been successful for *FMR1*-CGG loci, HD-CAG, etc.

Earlier efforts by Stevanovski *et al.* have successfully sequenced the STR region of the whole genome using ONT with ReadUntil API for live rejection of off-target sequencing reads. The approach followed by them has allowed to sequence all the STR regions of the human genome with a range of flanking regions analysis like haplotype, methylation, etc., highlighting the possible epigenetic roles in the pathogenic mechanism of the disorders. A long sequencing time span of 72 h and high molecular DNA input libraries with multiple reloads are required for obtaining optimal results, thus making the approach laborious and time-consuming.^24^

With the goal of targeted sequencing, LRseq of the amplified STR region using LR-PCR products makes the approach less laborious and sequencing can be performed in less time in a cost-effective manner. However, taking the advantages of ReadUntil API in account can help for making the further analysis smoother.

## Supplementary Material

fcad020_Supplementary_Data

## Data Availability

All data to support the findings of the present study are available in the paper and/or Supplementary material. Software used in the analysis is all free and open sources, except the proprietary ONT base-calling software Guppy. Raw sequencing data (FASTQ format) from the present study have been uploaded to Zenodo (https://doi.org/10.5281/zenodo.6958629). The commands for replicating the analysis workflow have been uploaded on Github (https://github.com/bharathramh/fxn_data).

## Abbreviations

AO =: Age of Onset
API =: Application Programming Interface
CAG =: Cytosine-Adenine-Guanine (trinucleotide repeat unit)
CGG =: Cytosine-Guanine-Guanine (trinucleotide repeat unit)
CTT =: Cytosine-Thymine-Thymine (trinucleotide repeat unit)
DTR =: Deep Tendon Reflex
ECHO =: Echocardiogram
EPS =: Electrophysiological Study
FFPE =: Formalin-Fixed, Paraffin-Embedded
FRDA =: Friedreich’s ataxia
GA =: Gait Ataxia
GAA =: Guanine-Adenine-Adenine (trinucleotide repeat unit)
GC =: Guanine Cytosine
HC =: Healthy Controls
HCM =: Hypertrophic Cardiomyopathy
HD =: Huntington’s Disease
HMM =: Hidden Markov Model
LRseq =: long-read sequencing
LR-PCR =: long-range polymerase chain reaction
LV =: Left Ventricle
LVH =: Left Ventricular Hypertrophy
NGS =: Next Generation Sequencing
NR =: Not recorded
OMIM =: Online Mendelian Inheritance of Man
ONT =: Oxford Nanopore Technologies
QC =: Quality Check
RP =: Repeat Primed
SCA =: Spinocerebellar Ataxia
SMN =: Sensory Motor Neuropathy
SN =: Sensory Neuropathy
SSR =: Sympathetic Skin Response
STR =: Short Tandem Repeats
TNR =: Tandem Nucleotide Repeats
