# Supplementary material for: Sequencing through hyperexpanded Friedreich’s ataxia-GAA repeats by nanopore technology: implications in genotype–phenotype correlation
Source: Brain Commun. 2023 Mar 29;5(2):fcad020. doi: 10.1093/braincomms/fcad020 (PMC10053634; doi:10.1093/braincomms/fcad020)

Sequencing through hyperexpanded FRDA-GAA repeats by Nanopore technology: Implication in genotype-phenotype correlation.

BharathramUppili[# Btech1,2] , Pooja Sharma[# MSc1,2], Istaq Ahmad [PhD3], Shweta Sahni [Msc3] Vivekananda A [MSc1,2], Anil BN [MD4], Achal K Srivastava [MD, DM3], Mohammed Faruq [MBBS, PhD 1,2#]*

Supplementary Information:

Primer sequence used in Long Range PCR:

Forward Primer- $^{5'}$GGAGGGATCCGTCTGGGCAAAGG$^{3'}$

Reverse Primer- $^{5'}$CAATCCAGGACAGTCAGGGCTTT$^{3'}$

Long Range PCR was performed using with KOD plus enzyme (Kit No 201 200U, 200 Reaction TOYOBO Com. Ltd Japan) with the following cycling conditions:

Initial Denaturation	No. of cycles=22	Final Extension
94℃ – 3minutes	94℃ – 20seconds	68℃- 10minutes
	68℃- 2.30minutes with 15seconds increment each cycle	

The formula used for the calculation of the GAA repeats after Long Range PCR:

No of GAA repeats= Amplified PCR Product size in base pairs - 1370 (Flanking region) / 3

Supplementary Fig. 1: Pedigree chart: Pedigree chart of family with cases of LOFA.

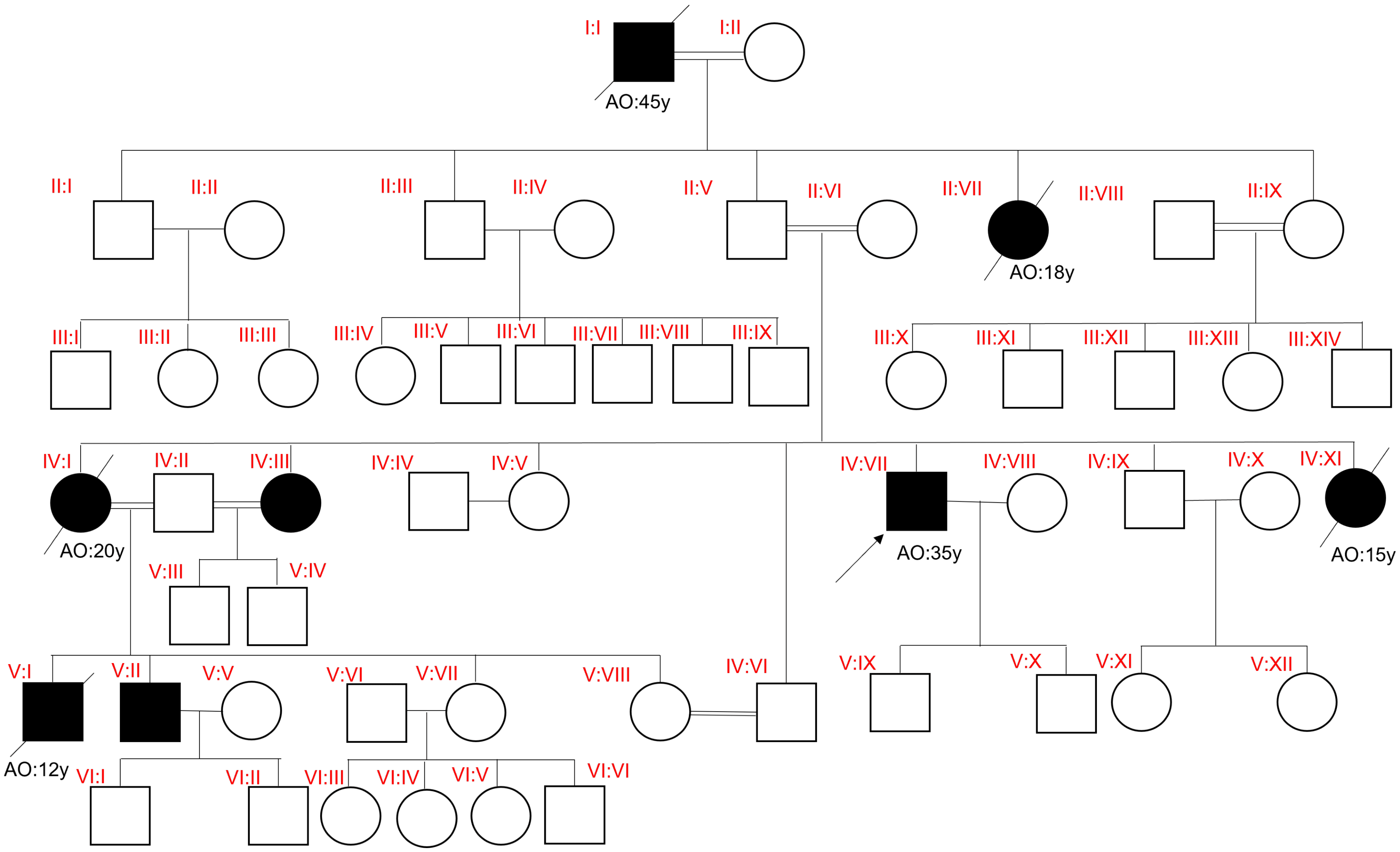

Supplementary Fig. 2: Long range-PCR base detection of GAA repeats and flanking region. A) the LR-PCR for kindred with late onset ataxia. B) Additional FRDA cases selected for LRseq.

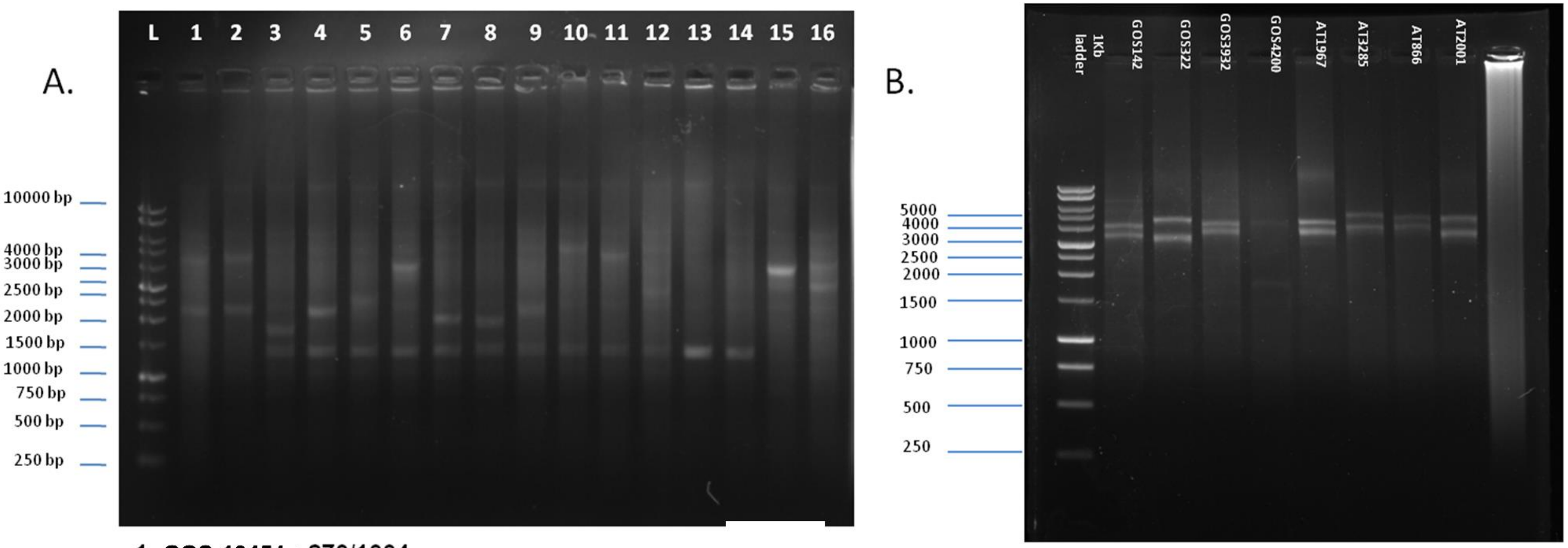

1. GOS-10454-: 270/1004
2. GOS-10455-: 287/1037
3. carrier.1-: 6/ 120
4. carrier.2-: 6/287
5. carrier.3-: 6/370
6. carrier.4-: 6/870
7. carrier.5-: 6/204
8. carrier.6-: 6/204
9. carrier.7-: 6/287
10. carrier.8-: 6/ 1370
11. carrier.9-: 6/ 1070
12. carrier.10-: 6/ 454
13. Healthy-: 6/ 6
14. Healthy-: 6/ 6
15. GOS-9443-: 870/870
16. patient4-: 537/ 870
17. L- 1000bp ladder

GOS142- GAA- 654/904
GOS322- GAA- 620/1054
GOS3932- GAA- 870/1204
GOS4200- GAA- 120/870
AT1967- GAA- 830/1200
AT3285- GAA-867/1200
AT866- GAA_866/1183
AT2001- GAA-704/1037

Supplementary Fig. 3: STRique squiggle representation of repeats: Current series pattern of representative reads in the form of squiggle for each of the FRDA samples.

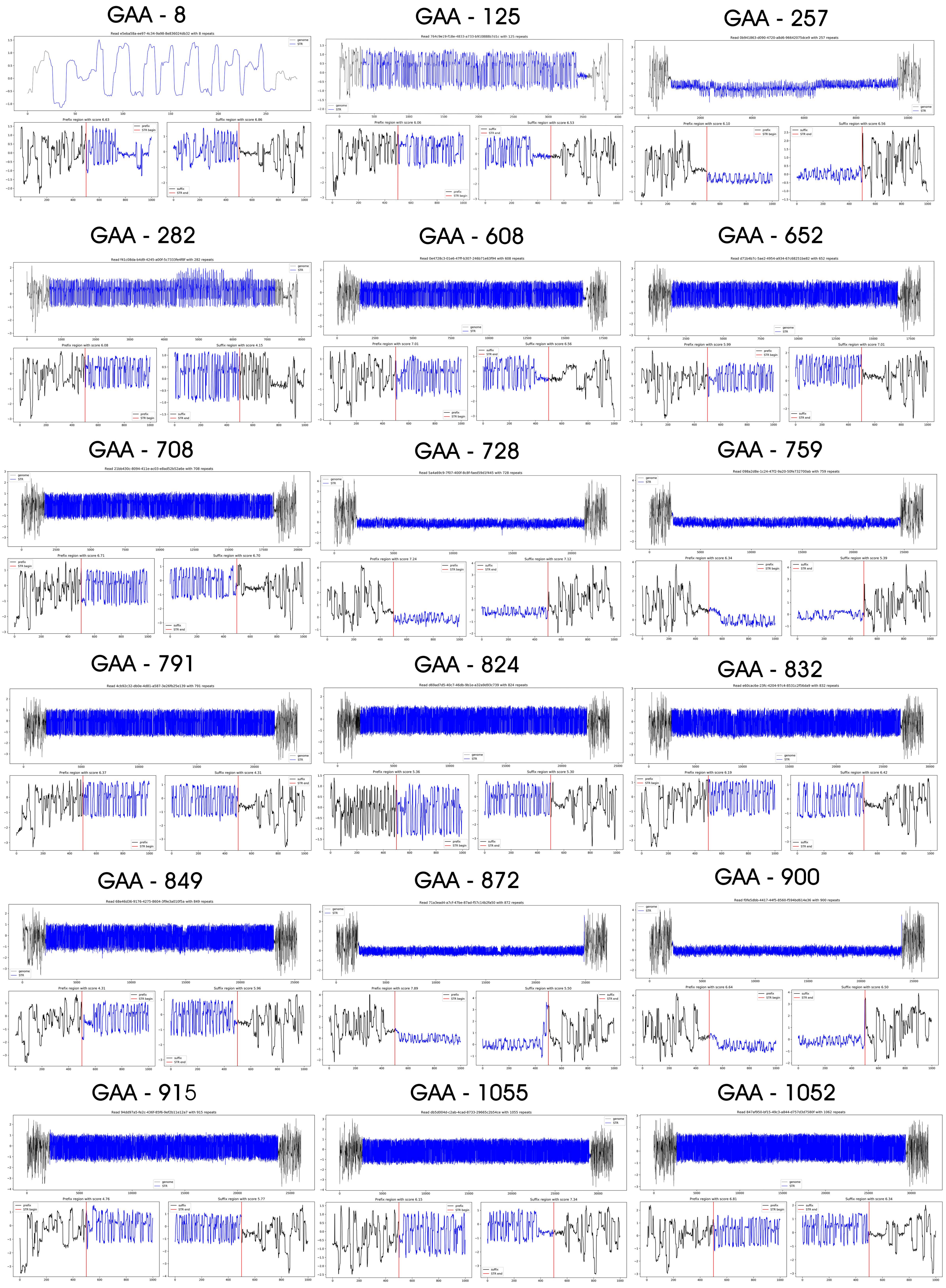

Supplement: fcad020_Supplementary_Data [file fcad020_supplementary_data.zip › Supplemantary_material.pdf]
